# Excluding external iliac node irradiation during neoadjuvant radiotherapy decreases lower intestinal toxicity without compromising efficacy in T4b rectal cancer patients with tumours involving the anterior structures

**DOI:** 10.1007/s12672-024-00885-6

**Published:** 2024-03-16

**Authors:** Anchuan Li, Miaobin Mao, Runfan Chen, Pan Chi, Ying Huang, Junxin Wu, Benhua Xu

**Affiliations:** 1 Department of Radiation Oncology, Fujian Medical University Union Hospital, Xinquan Road 29, Fuzhou, 350001 China; 2 Department of Radiation Oncology, College of Clinical Medicine, Fujian Medical University, Fuzhou, 350001 China; 3 Fujian Key Laboratory of Intelligent Imaging and Precision Radiotherapy for Tumors, Fujian Medical University, Fuzhou, 350001 China; 4 Clinical Research Center for Radiology and Radiotherapy of Fujian Province (Digestive, Hematological and Breast Malignancies), Fuzhou, 350001 China; 5 Department of Gastrointestinal Surgery, Fujian Medical University Union Hospital, Fuzhou, 350001 China; 6 Department of Radiation Oncology, Fujian Cancer Hospital, Fuma Road 420, Fuzhou, 350014 China

**Keywords:** Clinical target volume, External iliac node, Rectal cancer, Anterior structures, Radiotherapy

## Abstract

**Purpose:**

To explore the impact of excluding the external iliac node (EIN) from the clinical target volume (CTV) during preoperative radiotherapy in T4b rectal cancer with anterior structure invasion.

**Methods:**

We retrospectively identified 132 patients with T4b rectal cancer involving the anterior structures who received radiotherapy followed by surgery between May 2010 and June 2019. Twenty-nine patients received EIN irradiation (EIN group), and 103 did not (NEIN group). Failure patterns, survival and toxicities were compared between the two groups.

**Results:**

The most common failure was distant metastasis (23.5%). 11 (8.3%) patients developed locoregional recurrence, 10 (9.7%) patients were in the NEIN group, and 1 (3.4%) was in the EIN group (*P* = 0.34). The EIN region failure was rare (1/132, 0.8%). The locoregional recurrence-free survival (LRFS), distant metastasis-free survival (DMFS), overall survival (OS) and progression-free survival (PFS) rates were 96.3% vs. 90.5%, 82.1% vs.73.7%, 75.9% vs. 78.0% and 72.4% vs. 68.3% (all *P* > 0.05) for the EIN group and NEIN group, respectively. The incidence of grade 3–4 acute toxicity in the lower intestine was significantly higher in the EIN group than in the NEIN group (13.8% vs. 1.9%, *P* = 0.02). The Dmax, V35 and V45 of the small bowel was decreased in the NEIN group compared to the EIN group.

**Conclusions:**

Exclusion of the EIN from the CTV in T4b rectal cancer with anterior structure invasion could reduce lower intestinal toxicity without compromising oncological outcomes. These results need further evaluation in future studies.

**Supplementary Information:**

The online version contains supplementary material available at 10.1007/s12672-024-00885-6.

## Background

Colorectal carcinoma is the third most common cancer and has high morbidity and mortality worldwide [1]. Approximately 10% of rectal cancers either directly invade or adhere to contiguous organs at presentation [2] and are classified as T4b according to the Union for International Cancer Control/American Joint Committee on Cancer (UICC/AJCC) staging system. Preoperative chemoradiotherapy is an important treatment for patients with locally advanced rectal cancer and can improve the sphincter-preservation rate and lower the risk of recurrence [3–6]. However, acute gastrointestinal toxicity (notably diarrhoea) is the most common complication in patients receiving chemoradiotherapy, with grade 3 + diarrhoea occurring in approximately 11–39% of patients [3, 7]. Several studies have shown that the development of grade 3 diarrhoea was significantly associated with the volume of small bowel irradiated by all dose levels [8, 9]. Delineating a proper clinical target volume (CTV) for radiotherapy is important for avoiding exposure of the surrounding intestine to unnecessary doses and avoiding underdosing regions that have a high risk of recurrence.

Decisions on which areas should be included in the CTV must be evidence-based. For patients with T4b tumours involving the anterior structures, how much of the pelvic region should be contained in the radiation field remains unknown. Guidelines have recommended that the region of the external iliac node (EIN) should be included in the CTV for T4 tumours involving the anterior structures because of the lymphatic drainage patterns [10]. However, this recommendation was based on expert consensus and lacks related high-level clinical evidence. Previous studies have shown that EIN recurrence rarely occurs in rectal cancer patients, even in T4b patients [11, 12], but no studies limited to patients with invasion into the anterior organs were found in the literature. Likewise, no data regarding the necessity of elective irradiation of the EINs in this small cohort are available, and the irradiation of EINs remains open to debate.

Confirmatory evidence from long-term follow-up studies comparing patients with tumours involving the anterior structures who received radiotherapy with or without EIN irradiation is lacking. Thus, we conducted the present study to further explore the feasibility of excluding EIN irradiation in T4b rectal cancer patients with invasion into the anterior structures in this era of total mesorectal excision (TME) and modern treatment strategies.

## Methods

### Patient selection

Between May 2010 and January 2019, the hospital records of all rectal cancer patients who received preoperative radiotherapy or chemoradiotherapy followed by surgery at Fujian Medical University Union Hospital were reviewed. Consecutive patients with a radiological diagnosis of tumours involving the anterior structures (prostate, bladder, vagina, uterus, and seminal vesicle) were deemed eligible. The exclusion criteria were as follows: (1) external iliac lymph node swelling > 8 mm or abnormal 18F-fluorodeoxyglucose uptake at the initial presentation; (2) radiologically observed distant metastasis; (3) history of pelvic radiotherapy; and (4) incomplete medical information or treatment planning data.

Clinical staging and local tumour assessments were conducted, including physical examination, digital rectal examination, colonoscopy, chest computed tomography (CT), abdominal and pelvic magnetic resonance imaging (MRI) and endorectal ultrasonography. Additional positron emission tomography-computed tomography was performed. All patients underwent pelvic MRI examination at baseline, and all pelvic MRI images were retrospectively reviewed by two experienced radiologists to confirm the clinical stage. Tumour stage was assessed according to the 7th edition of the UICC/AJCC staging system.

The retrospective study was approved by the Clinical Research Ethics Committee of the Fujian Medical University Union Hospital. The patient records/information were anonymized and deidentified prior to analysis, and informed consent was not obtained from the participants due to the retrospective nature of the study.

### Radiotherapy

CT-based simulation scans were taken with patients in the supine position, with a 5 mm slice thickness. Patients were advised to follow an identical bladder filling protocol during CT-simulation scans and irradiation, which entailed bladder emptying followed by an oral fluid load of 500 ml of water and provide feedback on the bladder filling state. The gross tumour volume (GTV) was defined as the primary tumour and involved lymph nodes. The clinical target volume (CTV) was delineated according to the international consensus guidelines except for the delineation of the external iliac nodes. An isocentric margin of 5 mm was added to GTV or CTV to achieve the corresponding planning target volume (PTV). The CTV included all the macroscopic tumours with a minimum margin of 2 cm, the mesorectal and obturator regions, and the internal iliac and presacral nodes up to the L5/S1 junction. In the EIN group, additional EINs were delineated, while in the NEIN group, the EINs were excluded. The reduced irradiation range was due to a change in treatment policy at the department, with the intent to decrease toxicity. Radiotherapy was delivered with a minimum energy of 6-MV photons at 1.8 or 2.0 Gray (Gy) daily from Monday through Friday for a total of 25 to 28 fractions over 5 to 6 weeks and a total dose of 45 Gy to 50.4 Gy. All patients received preoperative radiotherapy with either 3-dimensional conformal radiotherapy (3D-CRT) or intensity-modulated radiotherapy (IMRT).

Organs at risk (OARs) included the small bowel, colon, urinary bladder, and femoral head. We contoured each small bowel loop for patients. All OARs were delineated to generate dose-volume histograms and maximum-tolerated doses and volumes. The recommended dose constraints were based on the QUANTEC review [13].

### Chemotherapy

The concurrent chemotherapy strategy consisted of oxaliplatin in combination with capecitabine (XELOX) or with 5-fluorouracil (FOLFOX/FOLFOX4), or capecitabine alone. The need for postoperative adjuvant chemotherapy was determined by the treating surgeon and the patient. Oxaliplatin-based regimens were the most commonly used.

### Toxicity and treatment plan analysis

Toxicity data during chemoradiotherapy were collected retrospectively from the medical records. The maximum toxicity grade was assigned using the Common Terminology Criteria for Adverse Events (version 4.0). Each patient’s radiotherapy treatment plan was reviewed, and dose-volume histograms were extracted for the small bowel, colon and bladder. The volumes of the structures (in absolute and relative numbers) that had received doses exceeding 5, 10, 15, 35 and 45 Gy were calculated (i.e., V5, V10, …, V45). The maximum dose to the structures (Dmax) and the dose by 30% of the volume (D30) or 50% of the volume (D50) were also recorded.

### Follow up

After finishing all treatment, patients were followed up every 3 months within the first 2 years, every 6 months for the following 3 years, and every year thereafter. The evaluations included physical examination, coloscopy, chest CT, abdominopelvic MRI and testing for gastrointestinal tumor markers. MRI was preferred for all patients, excepted if they declined an examination or had a contraindication for MRI. The abdominopelvic MRI was repeated 3 months after completion of treatment, and then semi-annually for three years and annually thereafter or earlier in cases of suspected recurrence. There were 15 disease-free living patients did not have pelvic CT or MRI done during the first 2 years of follow-up. Coloscopy and chest radiography was recommended at least once yearly.

### Statistical analysis

Oncological outcomes were evaluated by assessing locoregional recurrence-free survival (LRFS), distant metastasis-free survival (DMFS), overall survival (OS), and progression-free survival (PFS). LRFS was defined as the interval between the treatment commencement date and the first date of documented locoregional recurrence. Patients who died without locoregional recurrence were censored at the date of death for LRFS. DMFS was defined as the time of treatment commencement to the date of metastasis. OS was defined as the interval between the treatment commencement date and the date of death from any cause. PFS was measured from the treatment commencement date to the first date that any of the following events occurred: local and/or regional recurrence, distant metastasis, or death from any cause.

Patient clinical characteristics were summarized using descriptive statistics, and comparative analysis was performed using the χ^2^ test or Mann‒Whitney *U* test. Survival curves were estimated using the Kaplan‒Meier method and compared using the log-rank test. Multivariate analyses were performed using the Cox proportional hazards model (Cox regression). All statistical calculations were performed using *R* programming languages (*R* version 4.1.0). All tests used to explore statistical significance were 2-sided, and *P* < 0.05 was considered statistically significant.

## Results

### Patient characteristics

**Fig. 1:**
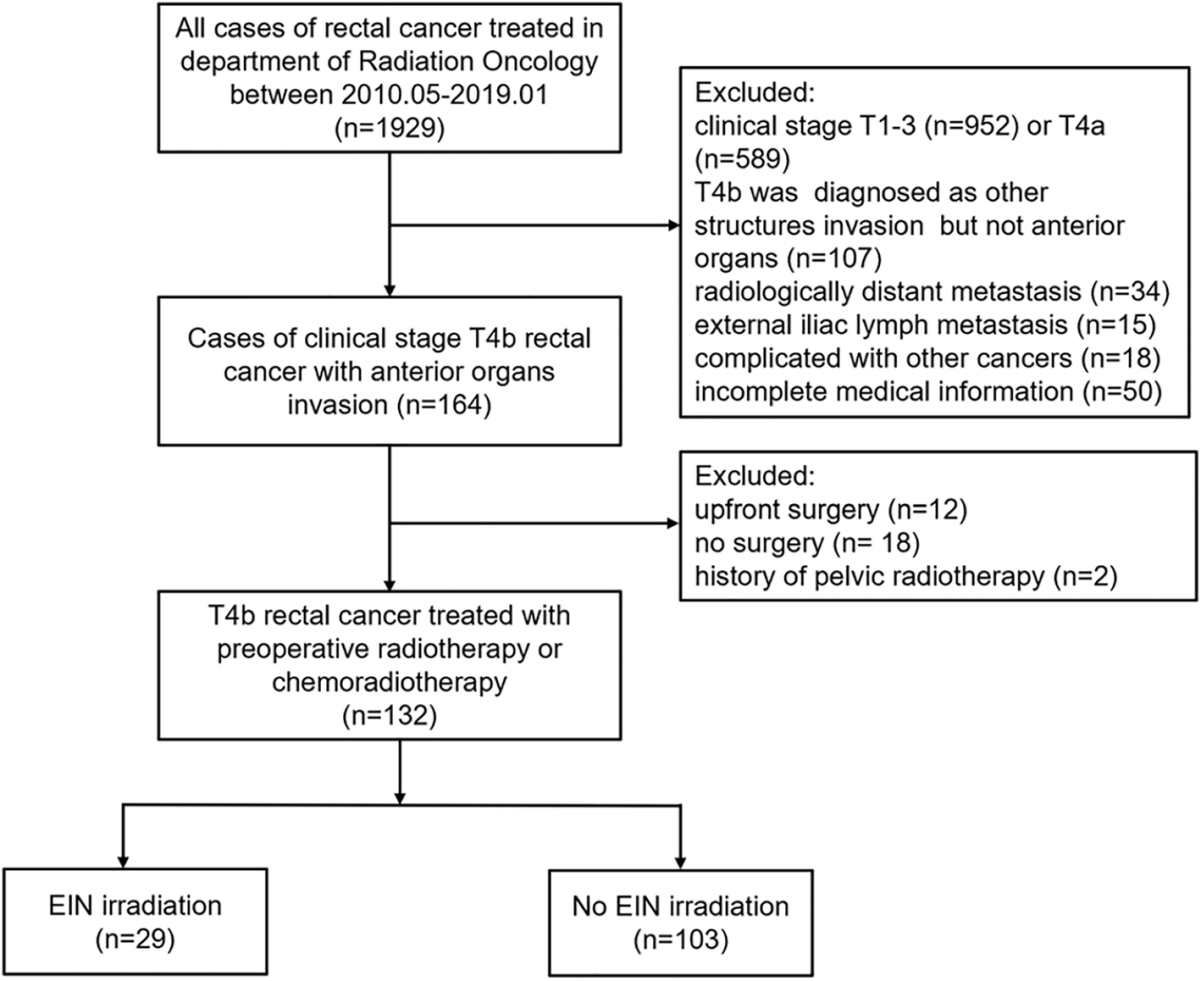
Included and excluded patients with rectal cancer

From May 2010 to January 2019, a total of 132 patients with T4b rectal cancer with tumours involving the anterior structures were included in the study; 29 patients (22.0%) were treated with EIN irradiation, and 103 (78.0%) were treated without EIN irradiation (see Fig. 1). The median age was 55 years (range, 24–81 years). A total of 90.9% of patients had tumours located in the mid-low rectum, and 94.7% were diagnosed with clinical lymph node metastasis. The clinical and pathological features of the two cohorts are compared in Table 1. The baseline characteristics were balanced between the two cohorts. Concurrent chemoradiotherapy was the most commonly used treatment strategy, and only four patients in the NEIN group did not receive chemotherapy, due to old age or poor health.

**Table 1:** Baseline clinical characteristics and treatment details of the rectal cancer patients

	EIN (n = 29)	NEIN (n = 103)	*P* value
Age (years) median (range)	56 (28–74)	55 (24–81)	0.963
Gender			
Male	11 (37.9%)	39 (37.9%)	
Female	18 (62.1%)	64 (62.1%)	1.000
Clinical stage			
II	2 (6.9%)	5 (4.9%)	
III	27 (93.1%)	98 (95.1%)	0.648
cN stage			
cN0	2 (6.9%)	5 (4.9%)	
cN1	2 (6.9%)	12 (11.7%)	
cN2	7 (24.1%)	43 (41.7%)	
cN1–2^a^	18 (62.1%)	43 (41.7%)	0.192
Distance from anal verge			
< 5 cm	11 (37.9%)	43 (41.7%)	
5–10 cm	15 (51.7%)	51 (49.5%)	
> 10 cm	3 (10.3%)	9 (8.7%)	0.862
Pretreatment CEA			
≤ 5 ng/ml	15 (51.7%)	48 (46.6%)	
> 5 ng/ml	14 (48.3%)	55 (53.4%)	0.626
Type of radiation			
3D-CRT	5 (17.2%)	16 (15.5%)	
IMRT	24 (82.8%)	87 (84.5%)	0.780
Concurrent chemotherapy			
No	0 (0.0%)	4 (3.9%)	
XELOX	6 (20.7%)	36 (35.0%)	
Capecitabine	22 (75.9%)	60 (58.3%)	
Other	1 (3.4%)	3 (2.9%)	0.293
Interval from RT to surgery (days), median (range)	63 (43–96)	68 (24–211)	0.106
Type of surgery			
Abdominoperineal resection	3 (10.3%)	11 (10.7%)	
Anterior resection	17 (58.6%)	68 (66.0%)	
Multivisceral resection	8 (27.6%)	21 (20.4%)	
Other	1 (3.4%)	3 (2.9%)	0.811
Pathological complete response			
Yes	2 (6.9%)	15 (14.6%)	
No	27 (93.1%)	88 (85.4%)	0.361

EIN, group with external iliac nodes radiation; NEIN, group without external iliac nodes radiation; 3D-CRT, 3-dimensional conformal radiotherapy; IMRT, intensity modulated radiotherapy; RT, radiotherapy

^a^Patients had lymph node metastasis but N stage could not be determined

### Treatment outcomes

The median follow-up was 60.5 months (range, 5–120 months). The rates of pathological complete response (pCR) in the EIN group and NEIN group were 6.9% and 14.6% (*P* = 0.36), respectively. Among the 132 patients, 79 (59.8%) patients had poor tumour downstaging (ypT3-4), including 18 (62.1%) in the EIN group and 61 (59.2%) in the NEIN group (Table 2, *P* = 0.60). Eight patients developed distant metastasis during treatment. Negative surgical margins were not achieved in 6 patients, and most occurred in the circumferential resection margin (CRM, 5/6). The CRM positivity rate was 3.8% in the whole cohort, with 4.9% in the NEIN group, and no patients in the EIN group had CRM positivity (Table 2).

**Table 2:** Pathologic outcomes of the rectal cancer patients

	EIN (n = 29)	NEIN (n = 103)	*P* value
ypT stage			
ypT0	2 (6.9%)	15 (14.6%)	
ypT1-2	9 (31.0%)	27 (26.2%)	
ypT3	11 (37.9%)	52 (50.5%)	
ypT4	7 (24.1%)	9 (8.7%)	0.094
ypN stage			
ypN0	19 (65.5%)	70 (68.0%)	
ypN1	6 (20.7%)	24 (23.3%)	
ypN2	4 (13.8%)	9 (8.7%)	0.677
ypStage			
0	2 (6.9%)	15 (14.6%)	
I	7 (24.1%)	21 (20.4%)	
II	9 (31.0%)	32 (31.1%)	
III	9 (31.0%)	29 (28.2%)	
IV	2 (6.9%)	6 (5.8%)	0.858
No. of sampled lymph nodes			
Median (range)	13 (0–30)	13 (0–42)	0.748
Surgical margin			
R0	29 (100.0%)	96 (93.2%)	
R1	0 (0.0%)	6 (5.8%)	0.483
CRM			
Negative	29 (100.0%)	96 (93.2%)	
Positive	0 (0.0%)	5 (4.9%)	0.749
Undetermined	0 (0.0%)	2 (1.9%)	
pCR			
Yes	2 (6.9%)	15 (14.6%)	
No	27 (93.1%)	88 (85.4%)	0.361
RCRG			
1	10 (34.5%)	47 (45.6%)	
2	14 (48.3%)	46 (44.7%)	
3	4 (13.8%)	10 (9.7%)	
Undetermined	1 (3.4%)	0 (0.0%)	0.241

EIN, group with external iliac nodes radiation; NEIN, group without external iliac nodes radiation; CRM, circumferential resection margin; pCR, pathological complete response; RCRG, rectal cancer regression

At the time of analysis, 35 patients (26.5%) were found to suffer from treatment failure.Distant failure occurred as the only site of failure in 24 patients (18.2%), while overall distant metastasis events were noted in 31 patients (23.5%), none of patients occurred distant metastases before local recurrence;locoregional recurrence occurred as the only site of failure in 4 patients (3.0%), and 1 patient (0.8%) suffered from distant metastasis nearly 2 years after local recurrence, 6 patients (4.5%) experienced locoregional recurrence and synchronous distant metastasis, and the total cumulative locoregional recurrence rate was 8.3%.

Ten out of 103 patients (9.7%) in the NEIN group developed locoregional failure, while in the EIN group, 1 patient (3.4%) experienced locoregional recurrence (*P* = 0.34). The features of the patients who developed locoregional recurrence are shown in Table 3. The median recurrence time was 28 months (7–92 months). Six patients had distant metastasis at the time of recurrence, while 1 patient experienced liver metastasis 21 months after regional recurrence. All 11 patients who developed locoregional recurrence were diagnosed as cN positive at baseline, and 7 patients had poor tumour downstaging (ypT3-4) after chemoradiotherapy. A greater proportion of locoregional recurrence events occurred in patients with lower rectal cancers than in those with mid-upper rectal cancers (11.1% vs. 6.4%).

**Table 3:** Features of the 11 patients with locoregional recurrence

Characteristics	Patient 1	Patient 2	Patient 3	Patient 4	Patient 5	Patient 6	Patient 7	Patient 8	Patient 9	Patient 10	Patient 11
Age	51	34	49	24	53	45	69	62	81	60	48
Sex	Male	Female	Male	Female	Male	Female	Female	Female	Female	Female	Female
EIN radiation	No	No	No	No	No	No	No	Yes	No	No	No
Clinical stage	T4bN +	T4bN +	T4bN +	T4bN2	T4bN2	T4bN2	T4bN +	T4bN +	T4bN +	T4bN +	T4bN +
Anterior organ involvement	Seminal vesicle	Vagina	Prostate	Uterus	Prostate	Uterus	Uterus	Uterus	Uterus	Uterus	Vagina
Tumor location (cm)	4	4.5	4.2	10.1	2.8	9.1	4.18	5.2	10	6	3.58
Concurrent chemotherapy	XELOX	Capecitabine	Capecitabine	XELOX	XELOX	No	Capecitabine	Capecitabine	Capecitabine	Capecitabine	Capecitabine
Surgery	LAR	LAR	LAR	LAR	Local excision	LAR	MVR	LAR	LAR	MVR	LAR
Number of retrieved lymph nodes	11	42	17	17	0	21	13	10	11	8	24
ypT stage	3	2	0	2	2	3	4b	3	3	4b	3
ypN stage	2a	1a	0	1a	NA	1a	1b	0	2a	2b	0
RCRG	1	2	1	1	1	2	2	1	3	2	2
Resection margin	R0	R0	R0	R0	R0	R0	R0	R0	R0	R1	R0
Positive CRM (pathological)	No	No	No	No	No	No	No	No	No	Yes	No
Positive EMVI (pathological)	Yes	No	No	No	No	Yes	Unknow	Unknow	Unknow	Yes	No
Recurrence time (month)	92	23	34	23	28	7	31	23	39	13	28
Recurrence site	Anastomotic	Anastomotic	Presacral space	Anastomotic	Anastomotic	Internal iliac lymph nodes	Bladder	Anastomoticsalpingo, bladder	Bladder	Bladder, perineum, internal iliac lymph nodes	External iliac lymph nodes
Distant metastasis	No	No	No	Yes	No	Yes	Yes	Yes	Yes	Yes	Yes
Distant metastasis time (month)	NA	NA	NA	23	NA	28	31	23	39	13	28

LAR, low anterior resection; MVR, multivisceral resection; RCRG, rectal cancer regression grade; CRM, circumferential resection margin; EMVI, extramural vascular invasion; NA, not applicable

All of the locoregional recurrences were below L5/S1 (Additional file 1: Fig. S2); 5 involved the anastomosis, 4 involved the bladder, 1 involved the presacral space, 2 involved the internal iliac lymph nodes, 1 involved the fallopian tubes, and 1 involved the perineum. One patient with a lower rectal tumour diagnosed as cT4bN2 (invaded the vagina) moderately differentiated adenocarcinoma developed EIN failure (unifocal failure). He received capecitabine-based neoadjuvant chemoradiotherapy (NCRT) without EIN irradiation, and the tumours were downstaged to ypT3N0 after NCRT. This resulted in a recurrence rate of 0.8% in all patients and 1.0% in the NEIN group. This patient was also diagnosed with supraclavicular and paraaortic lymph node metastasis at the time of EIN recurrence.

**Fig. 2:**
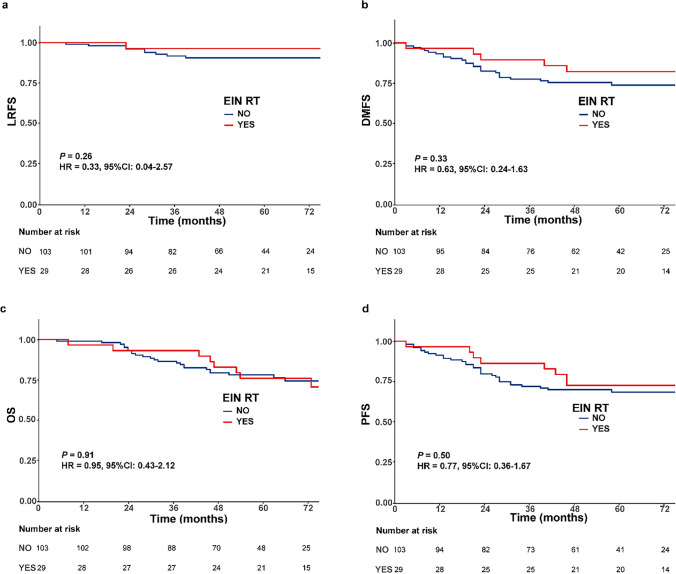
Kaplan–Meier survival curves of all patients treated with and without external iliac node (EIN) irradiation. **a** locoregional recurrence-free survival; **b** distant metastasis-free survival; **c** overall survival; **d** progression-free survival

We compared patient survival outcomes between the EIN group and the NEIN group. The 5-year LRFS, DMFS, OS and PFS were 96.3% vs. 90.5% (*P* = 0.26), 82.1% vs. 73.7% (0.33), 75.9% vs. 78.0% (*P* = 0.91) and 72.4% vs. 68.3% (*P* = 0.50) for the EIN group and NEIN group (Fig. 2). For patients with clinical lymph node metastasis, all survival outcomes remained similar and nonsignificant between the two groups (Additional file 1: Fig. S1).

Univariate and multivariate analyses demonstrated that EIN irradiation did not affect patients’ LRFS (*P* = 0.261, hazard ratio (HR) = 0.306, 95% confidence interval (CI) 0.039–2.412), DMFS (*P* = 0.301, HR = 0.602, 95% CI 0.230–1.575), OS (*P* = 0.672, HR = 0.840, 95 %CI 0.375–1.882) or PFS (*P* = 0.311, HR = 0.669, 95% CI 0.307–1.456) (Table 4).

**Table 4:** Univariate and multivariate analyses for treatment outcomes

	Univariate	Multivariate	
	*P* value	Hazard ratio (95%CI)	*P* value
LRFS			
Gender (female vs. male)	0.452	0.638 (0.166–2.455)	0.513
Age at diagnosis (≤ 55 vs. > 55 years)	0.439	0.611 (0.172–2.171)	0.446
Pretreatment CEA (≤ 5 vs. > 5 ng/ml)	0.789	1.120 (0.337–3.722)	0.854
Pretreatment CA199 (≤ 37 vs. > 37 ku/l)	0.173	2.314 (0.671–7.983)	0.184
EIN radiation (no vs. yes)	0.262	0.306 (0.039–2.412)	0.261
ypT stage (ypT0-2 vs. ypT3-4)	0.875	0.892 (0.313–2.540)	0.778
ypN stage (ypN0 vs. ypN1-2)	0.009	4.489 (1.311–15.368)	0.017
DMFS			
Gender (female vs. male)	0.135	0.536 (0.239–1.201)	0.130
Age at diagnosis (≤ 55 vs. > 55 years)	0.616	0.582 (0.284–1.191)	0.139
Pretreatment CEA (≤ 5 vs. > 5 ng/ml)	0.165	1.461 (0.685–3.113)	0.326
Pretreatment CA199 (≤ 37 vs. > 37 ku/l)	0.289	1.110 (0.494–2.494)	0.801
EIN radiation (no vs. yes)	0.333	0.602 (0.230–1.575)	0.301
ypT stage (ypT0-2 vs. ypT3-4)	< 0.001	5.875 (1.941–17.782)	0.002
ypN stage (ypN0 vs. ypN1-2)	0.007	1.602 (0.785–3.269)	0.195
OS			
Gender (female vs. male)	0.580	1.225 (0.600–2.501)	0.577
Age at diagnosis (≤ 55 vs. > 55 years)	0.710	1.065 (0.515–2.200)	0.866
Pretreatment CEA (≤ 5 vs. > 5 ng/ml)	0.256	1.173 (0.568–2.420)	0.666
Pretreatment CA199 (≤ 37 vs. > 37 ku/l)	0.068	1.656 (0.798–3.435)	0.176
EIN radiation (no vs. yes)	0.905	0.840 (0.375–1.882)	0.672
ypT stage (ypT0-2 vs. ypT3-4)	0.006	2.257 (1.062–4.796)	0.034
ypN stage (ypN0 vs. ypN1-2)	0.005	1.942 (0.959–3.935)	0.065
PFS			
Gender (female vs. male)	0.507	0.774 (0.399–1.500)	0.448
Age at diagnosis (≤ 55 vs. > 55 years)	0.658	0.797 (0.423–1.500)	0.482
Pretreatment CEA (≤ 5 vs. > 5 ng/ml)	0.811	0.923 (0.487–1.749)	0.806
Pretreatment CA199 (≤ 37 vs. > 37 ku/l)	0.168	1.468 (0.745–2.890)	0.267
EIN radiation (no vs. yes)	0.504	0.669 (0.307–1.456)	0.311
ypT stage (ypT0-2 vs. ypT3-4)	0.005	2.008 (1.062–3.796)	0.032
ypN stage (ypN0 vs. ypN1-2)	< 0.001	2.187 (1.148–4.164)	0.017

LRFS, locoregional recurrence-free survival; DMFS, distant metastasis-free survival; OS, overall survival; PFS, progress-free survival; EIN, external iliac node

### Toxicity and dosimetric parameters

We evaluated acute radiation-related toxicities at the time when toxic and side effects were most obvious during radiotherapy. The incidences of lower intestinal toxicity, radiodermatitis, and urinary toxicity were similar between the EIN group and the NEIN group. However, when considering severe toxicity, the NEIN group showed a significantly lower incidence of grade 3–4 diarrhoea than the EIN group (1.9% vs. 13.4%, *P* = 0.02). No cases of grade 3 + skin and urinary toxicity or treatment-related deaths occurred.

The median CTV in the NEIN group was 411 cm^3^ (range 188.8–1077.1 cm^3^), which was significantly smaller than that in the EIN group (521.9 cm^3^, range 240.9–871.2, *P* = 0.01). The specific dosimetric parameters are shown in Table 5. The Dmax of the small bowel was significantly decreased in the NEIN group compared to that in the EIN group (4779 vs. 5039 cGy, *P* < 0.01). The average volumes of small bowel irradiated with 35 and 45 Gy were significantly smaller in the NEIN group than in the EIN group, while the low-dose exposure volumes (V5, V10, V15) were not significantly different between the two groups. The V45 of the colon was consistently decreased in the patients treated without EIN irradiation compared to that in patients who received EIN irradiation.

**Table 5:** Dose volume parameters of the rectal cancer patients

	EIN	NEIN	*P* value
	Median (range)	Median (range)	
GTV (cm^3^)	64.3 (18.9–245.3)	61.2 (22.0–418.9)	0.831
CTV (cm^3^)	521.9 (240.9–871.2)	411.0 (188.8–1077.1)	0.011
Small bowel			
Dmax (cGy)	5039 (2904–5345)	4779 (309–5392)	0.001
V5 (cm^3^)	240.6 (14.1–531.8)	262.4 (0.0–696.20)	0.831
V10 (cm^3^)	212.4 (1.8–497.6)	239.4 (0.0–611.7)	0.831
V15 (cm^3^)	201.2 (0.1–460.0)	197.8 (0.0–585.2)	0.831
V35 (cm^3^)	91.1 (0.0–276.2)	45.8 (0.0–196.3)	0.003
V45 (cm^3^)	51.0 (0.0–130.0)	11.4 (0.0–116.6)	< 0.001
Colon			
Dmax (cGy)	5194 (3039–5428)	4944 (2630–5419)	0.286
V5 (cm^3^)	116.5 (40.0–462.9)	135.2 (26.2–830.7)	0.286
V10 (cm^3^)	102.8 (19.7–418.4)	120.8 (24.4–694.6)	0.286
V15 (cm^3^)	90.7 (17.6–411.7)	113.7 (20.8–645.5)	0.286
V35 (cm^3^)	55.5 (0.0–257.9)	47.1 (0.0–239.2)	0.135
V45 (cm^3^)	36.5 (0.0–109.0)	22.1 (0.0–219.7)	0.006
Bladder			
D30 (cGy)	4483 (2428–5145)	3752 (1296–5182)	< 0.001
D50 (cGy)	3938 (2161–4764)	3205 (1090–5143)	< 0.001
V45 (cm^3^)	29.0 (0–117.3)	11.8 (0.0–87.9)	0.001

EIN, group with external iliac nodes radiation; NEIN, group without external iliac nodes radiation; GTV, gross tumour volume, including only the primary tumour volume; CTV, clinical target volume

## Discussion

The present study was the first to focus on T4b patients with tumours invading the anterior organs. The study yielded three main findings. First, the rate of locoregional recurrence was low for T4b patients with invasion into the anterior structures (8.3%), and EIN failure was rare (1/132). Distant metastasis was the main failure pattern in patients after multimodality therapy. Second, oncologic outcomes (LRFS, DMFS, OS and PFS) were similar between the EIN group and NEIN group, both in the full cohort and in the patients with clinical lymph node metastasis. The omission of EIN irradiation was not a significant risk factor impacting LRFS, DMFS, OS or PFS. Thus, reducing the CTV by excluding EIN irradiation is safe in patients with tumours involving the anterior structures regardless of N stage. Finally, in comparison with EIN irradiation, treatment excluding EIN irradiation resulted in less severe lower intestinal toxicity. Exclusion of the EINs from the CTV decreased the volumes of the small bowl and colon exposed to a high dose, which were thought to be factors related to lower intestinal toxicity.

With advances in the multidisciplinary management of rectal cancer, the local recurrence rate has significantly decreased to below 10% in locally advanced rectal cancer, and distant metastasis remains the main cause of treatment failure [14–16]. In the present study, the cumulative incidence of distant metastasis for T4b rectal cancer with anterior organ invasion was 23.5%, compare with 8.3% of locoregional recurrence, consistent with other studies investigating T4 rectal cancer; this shows that distant recurrence is substantially more common than local recurrence in patients with T4 tumours [11, 16–18]. T4b tumours penetrate to directly invade adjacent organs or structures, which is already an indication of the dissemination of tumour cells. This partly explained the high rates of distant metastasis in T4b rectal cancers. However, the locoregional recurrence rate of 8.3% in our study was lower than that in the studies mentioned above (12.5–23.5%). A possible reason is that the positive CRM rate in our study was relatively low (3.8%) compared to the rate of 10.7–35.2% reported by previous studies [16, 18, 19].

The EIN region is not a primary nodal drainage pathway for rectal cancer, so it is not routinely included in the radiation field, but when the tumour involves the anterior structures, the consensus panel agreed that the EINs should be added based on the pattern of lymphatic drainage appropriate for the gynaecologic or genitourinary system [20]. However, no evidence has shown that the real biological behaviour of tumours originating in the rectum is consistent with that of tumours originating in the gynaecology or genitourinary system. In the present study, the EIN failure rate was low (0.8%, 1/132) in rectal cancer patients with anterior organ invasion, and no difference was observed between the patients treated with EIN irradiation and those treated without EIN irradiation. Similar findings were reported by several studies. A study from the MD Anderson Cancer Center included 45 patients with T4b rectal cancer, all of whom received NCRT without EIN irradiation, and no nodal recurrence was found in the EIN region [11]. However, the study was performed in earlier years, and at that time, tumour stage was evaluated based on CT, which is considered to be less accurate than MRI. Recently, Zhang et al. omitted EIN irradiation in T4b patients and reported an EIN failure rate of 0.8% among all T4b patients and 1.8% in the group with anterior genitourinary organ invasion [12]. Several studies have reported different results. The analysis of locoregional relapses in the ACCORD12/0405-PRODIGE 02 trial showed that 6.5% of patients with T4 tumours had recurrence in the external iliac or anterior lateral lymph nodes, although those patients were treated with EIN irradiation [21]. This analysis was based on a small sample size, there were only 31 patients with T4 tumours in the trial, and bias might arise as large random fluctuations of the estimated treatment effect could occur. We did not find that EIN irradiation affected the treatment outcome. The EIN and NEIN groups had the same rates of LRFS, DMFS, OS and PFS, and for patients diagnosed with clinical lymph node metastasis, NEIN irradiation consistently did not decrease the LRFS, DMFS, OS and PFS rates compared to EIN irradiation. In the multivariate analysis, EIN irradiation failed to be a significant prognostic factor for LRFS, DMFS, OS or PFS. Based on the results and the above studies, it may be unnecessary to extend the CTV to include the area of the EINs. Further prospective clinical trials involving a larger cohort are needed.

Previous studies reported that the omission of EIN irradiation led to a low rate of severe lower intestinal toxicity in patients with T4 rectal cancer [11, 12]. Similarly, our study observed a lower incidence of acute grade 3–4 diarrhoea in the NEIN group than in the EIN group (1.9% vs. 13.8%, *P* = 0.02). This may be explained by the reduction in the CTV from excluding EIN irradiation, resulting in a reduction in the dose–volume parameters of the small bowel and colon. The average volume of the small bowel irradiated by each 5 Gy dose level from 5 to 40 Gy was reported to be associated with the development of grade 3 acute diarrhoea by several studies [9, 22, 23]. Robertson reported that grade 3 diarrhoea typically occurred at a median dose of 30.6 Gy with a range of 12.6–43.2 Gy [9]. In the present study, the Dmax, V35 and V45 of the small bowel were significantly lower in the NEIN group than in the EIN group, which explained the lower incidence of grade 3 diarrhoea in the NEIN group.

Several limitations to this study need to be considered. First, this was a retrospective comparison, and this study only included patients from a single centre. Interpretation of the results is somewhat limited by the degree of heterogeneity. Second, the small sample size limited the statistical power of the analyses; however, to our knowledge, this is the largest study that has focused on CTV delineation in T4b patients with anterior organ invasion. Thus, in the absence of prospective studies, this report adds further data to the paucity of literature on CTV delineation for patients with tumours involving the anterior structures.

## Conclusions

In conclusion, EIN recurrence was rare in T4b rectal cancer patients with tumours involving the anterior structures who were treated without EIN region irradiation. Exclusion of the EIN from the CTV during preoperative radiotherapy yielded similar survival outcomes to treatment with EIN irradiation and was associated with less acute and less severe lower intestinal side effects. These results need further evaluation in future randomized trials.

### Supplementary Information

**Additional file 1: Figure S1.** Kaplan–Meier survival curves of patients with cN positivity treated with and without EIN irradiation. **a** locoregional recurrence-free survival; **b** distant metastasis-free survival; **c** overall survival; **d** progression-free survival. **Figure S2.** Schematic diagram of sites of locoregional recurrence.

## Data Availability

The datasets used during the current study are available from the corresponding author on reasonable request.

## Abbreviations

EIN: External iliac node
NEIN: Not external iliac node
CTV: Clinical target volume
LRFS: Locoregional recurrence-free survival
DMFS: Distant metastasis-free survival
OS: Overall survival
PFS: Progression-free survival
UICC/AJCC: Union for International Cancer Control/American Joint Committee on Cancer
TME: Total mesorectal excision
CT: Chest computed tomography
MRI: Abdominal and pelvic magnetic resonance imaging
GTV: Gross tumor volume
3D-CRT: 3-Dimensional conformal radiotherapy
IMRT: Intensity modulated radiotherapy
OARs: Organs at risk
pCR: Pathological complete response
CRM: Circumferential resection margin
NCRT: Neoadjuvant chemoradiotherapy
HR: Hazard ratio
CI: Confidence interval
TRG: Tumor regression grade
