# Supplementary material for: Excluding external iliac node irradiation during neoadjuvant radiotherapy decreases lower intestinal toxicity without compromising efficacy in T4b rectal cancer patients with tumours involving the anterior structures
Source: Discov Oncol. 2024 Mar 16;15:76. doi: 10.1007/s12672-024-00885-6 (PMC10944434; doi:10.1007/s12672-024-00885-6)
Supplement: Supplementary file 1 — Additional file 1: Figure S1. Kaplan–Meier survival curves of patients with cN positivity treated with and without EIN irradiation. a locoregional recurrence-free survival; b distant metastasis-free survival; c overall survival; d progression-free survival. Figure S2. Schematic diagram of sites of locoregional recurrence. [file 12672_2024_885_MOESM1_ESM.pdf]

Excluding external iliac node irradiation during neoadjuvant radiotherapy decreases lower intestinal toxicity without compromising efficacy in T4b rectal cancer patients with tumours involving the anterior structures

Authors: Anchuan Li[1,2,3,4], Miaobin Mao[1,3,4]; Runfan Chen[1], Pan Chi[5]; Ying Huang[5]; Benhua Xu[1,2,3,4*]

[1] Department of Radiation Oncology, Fujian Medical University Union Hospital, Fuzhou, 350001, China;

[2] Department of Radiation Oncology, College of Clinical Medicine, Fujian Medical University, Fuzhou, 350001, China;

[3] Fujian Key Laboratory of Intelligent Imaging and Precision Radiotherapy for Tumors, Fujian Medical University, Fuzhou, 350001, China;

[4] Clinical Research Center for Radiology and Radiotherapy of Fujian Province (Digestive, Hematological and Breast Malignancies), Fuzhou, 350001, China;

[5] Department of Gastrointestinal Surgery, Fujian Medical University Union Hospital,

Fuzhou, 350001, China;

* **Corresponding author:** Ben-hua Xu, Department of Radiation Oncology, Fujian Medical University Union Hospital, Xinquan Road 29, Fuzhou, 350001, China; Tel: (86) 0591-86218731; Fax: (86) 0591-86218731; Email: benhuaxu@163.com.

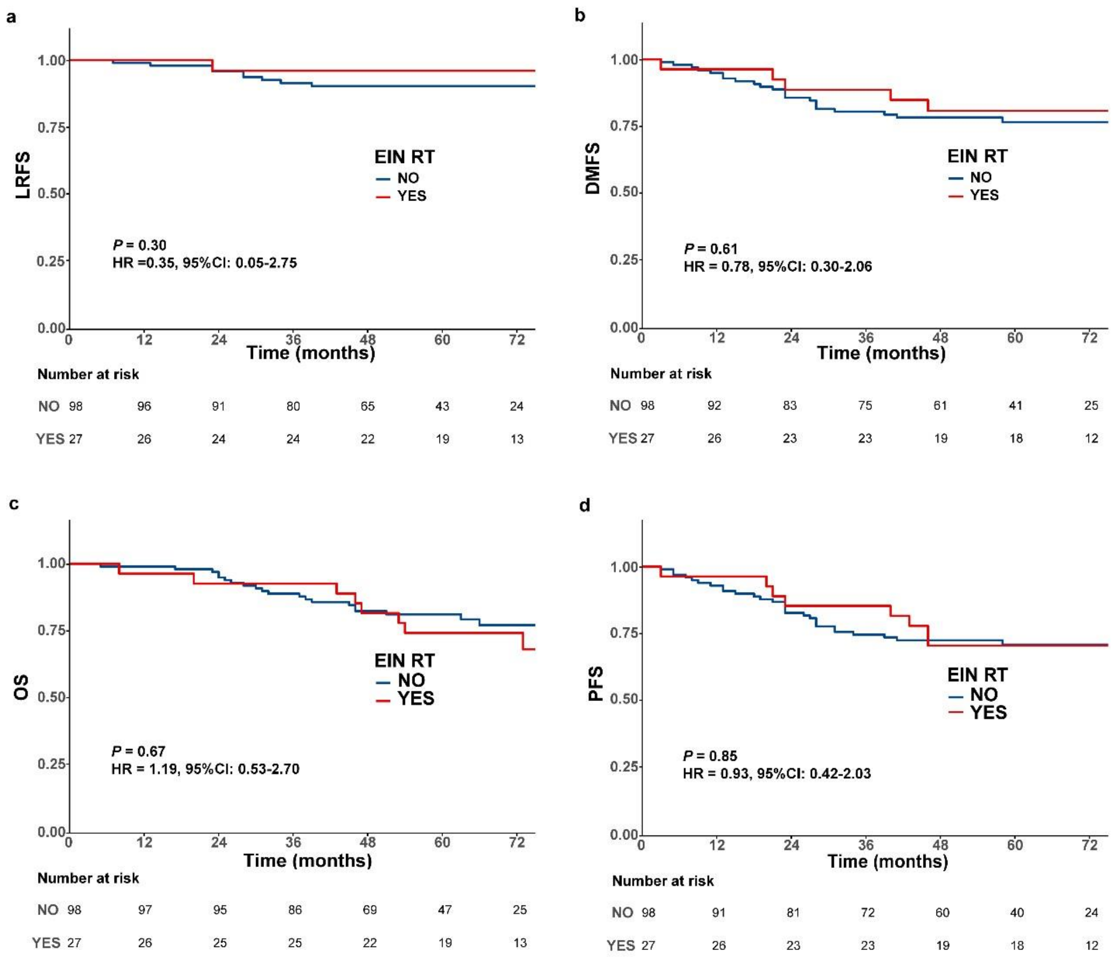

Figure S1 Kaplan–Meier survival curves of patients with cN positivity treated with and without EIN irradiation. A）locoregional recurrence-free survival; B) distant metastasis-free survival; C) overall survival; D) progression-free survival

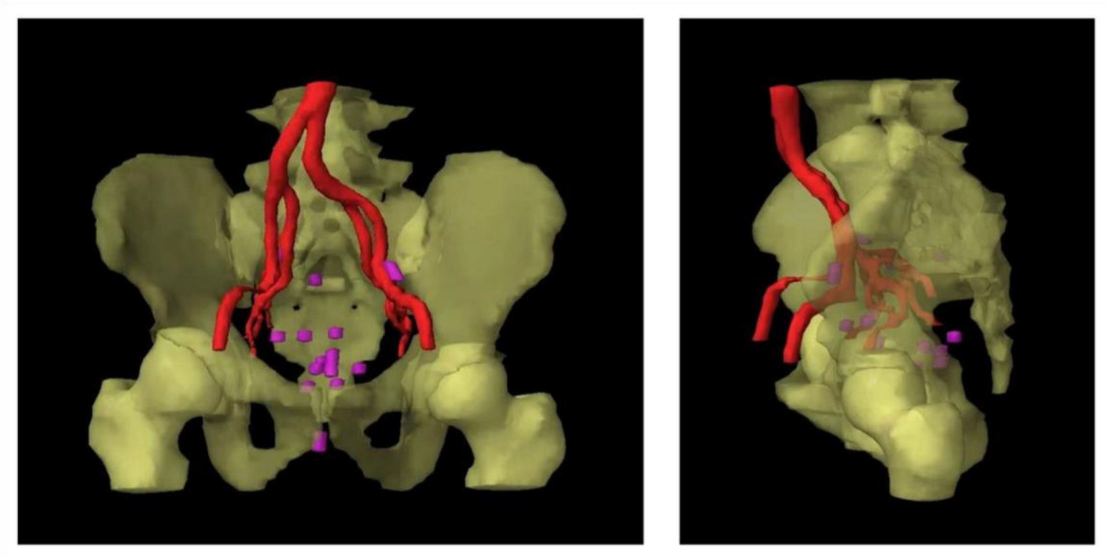

Figure S2 Schematic diagram of sites of locoregional recurrence
